# Supplementary material for: Prognostic Significance of the Extranodal Extension of Regional Lymph Nodes in Stage III-N2 Non-Small-Cell Lung Cancer after Curative Resection
Source: J Clin Med. 2021 Jul 28;10(15):3324. doi: 10.3390/jcm10153324 (PMC8347115; doi:10.3390/jcm10153324)
Supplement: Supplementary file 1 [file jcm-10-03324-s001.zip › jcm-1238735-supplementary.pdf]

STROBE Statement—Checklist of items that should be included in reports of ***cohort studies***

	Item No	Recommendation	Page, Line number (PDF version Manuscript)
**Title and abstract**	1	(*a*) Indicate the study's design with a commonly used term in the title or the abstract	Page 1, Line 2-4<br>Page 1, Line 20-26
		(*b*) Provide in the abstract an informative and balanced summary of what was done and what was found	Page 1, Line 28-33
**Introduction**			
Background/rationale	2	Explain the scientific background and rationale for the investigation being reported	Page 2, Line 3-18
Objectives	3	State specific objectives, including any prespecified hypotheses	Page 2, Line 18-23
**Methods**			
Study design	4	Present key elements of study design early in the paper	Page 2, Line 28-37
Setting	5	Describe the setting, locations, and relevant dates, including periods of recruitment, exposure, follow-up, and data collection	Page 2, Line 28-35<br>Page 4, Line 1-18
Participants	6	(*a*) Give the eligibility criteria, and the sources and methods of selection of participants. Describe methods of follow-up	Page 2, Line 28-33<br>Page 4, Line 1-18
		(*b*) For matched studies, give matching criteria and number of exposed and unexposed	Page 4, Line 26-29<br>Page 7, Line 12-17
Variables	7	Clearly define all outcomes, exposures, predictors, potential confounders, and effect modifiers. Give diagnostic criteria, if applicable	Page 2, Line 33-37<br>Page 2, Line 38-44<br>Page 3 (Figure 1)
Data sources/ measurement	8*	For each variable of interest, give sources of data and details of methods of assessment (measurement). Describe comparability of assessment methods if there is more than one group	Page 2, Line 38-44<br>Page 3 (Figure 1)
Bias	9	Describe any efforts to address potential sources of bias	Page 4, Line 26-29
Study size	10	Explain how the study size was arrived at	Page 2, Line 28-33
Quantitative variables	11	Explain how quantitative variables were handled in the analyses. If applicable, describe which groupings were chosen and why	Page 2, Line 33-35
Statistical methods	12	(*a*) Describe all statistical methods, including those used to control for confounding	Page 4, Line 19-32
		(*b*) Describe any methods used to examine subgroups and interactions	Page 4, Line 19-32
		(*c*) Explain how missing data were addressed	N/A, There were no missing data.
		(*d*) If applicable, explain how loss to follow-up was addressed	N/A, There was no patient with follow-up loss.
		(*e*) Describe any sensitivity analyses	N/A
**Results**			
Participants	13*	(a) Report numbers of individuals at each stage of study—eg numbers potentially eligible, examined for	Page 2, Line 28-33<br>Page 4, Line 35-36

		eligibility, confirmed eligible, included in the study, completing follow-up, and analysed	
		(b) Give reasons for non-participation at each stage	Page 2, Line 28-33
		(c) Consider use of a flow diagram	The patients included in the study is clear, so no diagram is necessary.
Descriptive data	14*	(a) Give characteristics of study participants (eg demographic, clinical, social) and information on exposures and potential confounders	Page 4, Line 36-47
		(b) Indicate number of participants with missing data for each variable of interest	There were no missing data.
		(c) Summarise follow-up time (eg, average and total amount)	Page 4, Line 35-36
Outcome data	15*	Report numbers of outcome events or summary measures over time	Page 4, Line 33 ~ Page 8, Line 3
Main results	16	(*a*) Give unadjusted estimates and, if applicable, confounder-adjusted estimates and their precision (eg, 95% confidence interval). Make clear which confounders were adjusted for and why they were included	Page 7, Line 12-20
		(*b*) Report category boundaries when continuous variables were categorized	N/A, Continuous variables were not arbitrarily changed to categorical variables.
		(*c*) If relevant, consider translating estimates of relative risk into absolute risk for a meaningful time period	N/A
Other analyses	17	Report other analyses done—eg analyses of subgroups and interactions, and sensitivity analyses	Page 7, Line 1-11
**Discussion**			
Key results	18	Summarise key results with reference to study objectives	Page 9, Line 31-36
Limitations	19	Discuss limitations of the study, taking into account sources of potential bias or imprecision. Discuss both direction and magnitude of any potential bias	Page 9, Line 21-29
Interpretation	20	Give a cautious overall interpretation of results considering objectives, limitations, multiplicity of analyses, results from similar studies, and other relevant evidence	Page 9, Line 31-36
Generalisability	21	Discuss the generalisability (external validity) of the study results	Page 9, Line 21-36
**Other information**			
Funding	22	Give the source of funding and the role of the funders for the present study and, if applicable, for the original study on which the present article is based	Page 9, Line 43

*Give information separately for exposed and unexposed groups.

Note: An Explanation and Elaboration article discusses each checklist item and gives methodological background and published examples of transparent reporting. The STROBE checklist is best used in conjunction with this article (freely available on the Web sites of PLoS Medicine at http://www.plosmedicine.org/, Annals of Internal Medicine at

http://www.annals.org/, and Epidemiology at http://www.epidem.com/). Information on the STROBE Initiative is available at http://www.strobe-statement.org.
